# Is intuition allied with jumping to conclusions in decision-making? An intensive longitudinal study in patients with delusions and in non-clinical individuals

**DOI:** 10.1371/journal.pone.0261296

**Published:** 2021-12-20

**Authors:** Thea Zander-Schellenberg, Sarah A. K. Kuhn, Julian Möller, Andrea H. Meyer, Christian Huber, Roselind Lieb, Christina Andreou

**Affiliations:** 1 Faculty of Psychology, Division of Clinical Psychology and Epidemiology, University of Basel, Basel, Switzerland; 2 Psychiatric University Hospital (UPK), University of Basel, Basel, Switzerland; 3 Department of Psychiatry and Psychotherapy, Translational Psychiatry Unit, University of Luebeck, Luebeck, Germany; National Institutes of Health, UNITED STATES

## Abstract

Research suggests that a jumping-to-conclusions (JTC) bias, excessive intuition, and reduced analysis in information processing may favor suboptimal decision-making, both in non-clinical and mentally disordered individuals. The temporal relationship between processing modes and JTC bias, however, remains unexplored. Therefore, using an experience sampling methodology (ESM) approach, this study examines the temporal associations between intuitive/analytical information processing, JTC bias, and delusions in non-clinical individuals and patients with schizophrenia. Specifically, we examine whether a high use of intuitive and/or a low use of analytical processing predicts subsequent JTC bias and paranoid conviction. In a smartphone-based ESM study, participants will be prompted four times per day over three consecutive days to answer questionnaires designed to measure JTC bias, paranoid conviction, and preceding everyday-life intuition/analysis. Our hierarchical data will be analyzed using multilevel modelling for hypothesis testing. Results will further elucidate the role of aberrant human reasoning, particularly intuition, in (non-)clinical delusions and delusion-like experiences, and also inform general information processing models.

## Introduction

Imagine Paula and Tom sitting together and planning their next holiday destination. Whereas Paula is comparing weather conditions, cultural offers, hotel prices and the like of different resorts, Tom simply goes with his gut feeling and chooses the destination that spontaneously feels best for him. After having chosen a specific destination, he will not be able to give rational reasons why he decided the way he did. In sum, Paula would make a list of pros and cons to extensively analyze the given alternatives, and Tom would decide rather intuitively.

In science, this differentiation of analytical versus intuitive information processing has been formalized in dual-process accounts of judgment and decision-making (see e.g., [1–5]). Intuitive Type 1 processes operate fast, affectively charged, holistically, and are based on associative memories, whereas analytical Type 2 processes are slower, effortful, intentional, and based on working memory and general cognitive capacity. In adaptive decision-making, both processes act jointly in order to guarantee conflict-free and flexible decision outcomes [3,6,7]. Thinking this through further, each mode of processing, taken by itself, has its distinct adaptive functions. Intuition, for instance, is associated with indicators of subjective wellbeing, such as experiencing meaning in life [8,9] and positive affect [10,11]. When in an intuitive mindset, people are able to spontaneously identify the essentials of the decision situation at hand by effectively integrating relevant information [12–14], feel connected to their true selves when making choices [15], adequately engage in parent-child interactions [16], and arrive at “smart” decisions under time pressure and with minimal cognitive effort [17–20]. In contrast, deliberate analysis is good for decision situations requiring extensive pondering and balancing pros and cons, and for accurate decision outcomes, for example, when solving laboratory tasks in accordance with the standard models of logic [21]. When engaging in analytical processing, people tend to perform better in lie detection [22], make everyday life decisions of self-rated and externally rated high quality [23], and are less prone to conjunction fallacies [24] and other deviations from logical reasoning such as framing effects [25].

People differ in their tendency to rely on intuition or analysis. Various validated trait questionnaires, such as the Rational Experiential Inventory (REI-40; [26]) or the Preference for Intuition/Deliberation scale (PID; [27]), have been created to capture these tendencies. Beside personal preferences [28–30] and certain characteristics of the decision situation that foster analysis or intuition (e.g., time pressure or complexity, especially in naturalistic decision-making; [31]), research suggests that also mental health conditions are intertwined with the applied processing mode [32,33]. In this context, disorders on the schizophrenia spectrum seem to be of prime interest. Research has shown that individuals suffering from schizophrenia exhibit certain reasoning abnormalities. For example, studies observed a lowered decision threshold in probabilistic reasoning tasks in this population (see [34] for a theoretical account). In this respect, many studies have robustly demonstrated that patients with schizophrenia arrive at a decision or judgment very early without gathering enough evidence to adequately process the information at hand–a phenomenon termed jumping-to-conclusions (JTC) bias (e.g., [35–38]). Moreover, reviews and meta-analyses have (a) demonstrated the existence of the JTC bias in patients with schizophrenia, especially in those who experience delusions, and in a less extreme form in healthy individuals, and (b) shown that the JTC bias distinguishes patients with schizophrenia from healthy and clinical controls [39–42]. As a result, various theoretical models on delusion formation and maintenance have incorporated the JTC bias as one potential (trait) vulnerability factor (e.g., [42,43]).

Recently, the JTC reasoning bias has been theoretically discussed in the context of dual-process accounts of judgment and decision-making [7,32,44,45]. In its classic form, these accounts propose a dynamic interaction between intuition and analysis as well as an enhanced error-proneness of intuitive Type 1 processes due to the human cognitive system, which tends to save effort whenever possible (e.g., [2,45]). In the field of delusion-associated reasoning biases, it can be surmised that, on the one hand, intuitive Type 1 processing is excessively active and/or that, on the other hand, analytical Type 2 processing is too reserved or even inactive and thus cannot override maladaptive Type 1 outcomes where necessary [32]. For example, in their Dual Stream Modulation Failure model, Speechley and Ngan [7] propose that delusions in schizophrenia arise as a result of a conflict modulation failure that prevents analytical Type 2 processes from effective functioning and correcting erroneous intuitive Type-1-based beliefs. In this respect, the JTC bias can be seen as one manifestation of an aberrant interaction between Type 1 and Type 2 processes, putatively resulting from an overreliance on maladaptive intuitive processes [32] that are unlikely to be corrected by Type 2 processes [7]. Assuming that the JTC bias forms one pillar in the creation of delusions, it can be argued that impaired analytical processes and an excessive and maladaptive use of intuitive processes engender a more pronounced JTC bias which, in turn, is linked to subsequent delusion-like or delusional beliefs. Importantly, both reasoning biases and delusions seem to be located on continuums which span slight aberrations in reasoning and non-pathological unusual experiences in the general population until severe reasoning biases and pathological delusions in individuals from the schizophrenia spectrum disorder. At the empirical level, a profound body of evidence has for instance demonstrated that both a JTC bias [39] and psychotic experiences [46,47] can be observed in non-clinical individuals, too, albeit to a lesser degree than in clinical individuals. Consequently, investigating the link between intuitive and analytical processes in relation to JTC bias is not only relevant for psychopathological models of delusions, but also for explanations of general judgment and decision-making models in healthy people. That is why, in our study, we start exactly at that point and aim to both contribute to a better understanding of delusion-related reasoning mechanisms in specific, and shed more light into the link between JTC bias and processing modes within hasty decision-making in general.

Supporting the notion of processing modes dovetailing with delusion-like beliefs, Freeman and colleagues [48], using a non-clinical sample, found small positive correlations between trait intuition preferences and persecutory ideation as well as small negative correlations between trait analysis preferences and persecutory ideation. Unfortunately, this pattern of high intuition preferences coexisting with lower levels of analysis preferences could not be replicated in a bigger follow-up study involving delusional patients with schizophrenia [49]. However, research in non-clinical samples observed a positive association of intuitive Type 1 processing with the occurrence of paranormal and superstitious beliefs and schizotypal traits in various student populations [50–54], and also with the occurrence of conspiracy theories [55,56], fake news [57], and pseudo-profound beliefs [58] in the general population. Moreover, experimental research found that intuitive processing (either laboratory-induced or naturally occurring as trait preference) can predict the belief in difficult-to-prove concepts like the belief in a God [59] and, in interaction with positive affect, also referential thinking [60] and even represent behavioral indicators of a leaning towards sympathetic magic [61]. Furthermore, a study [62] observed that intuitive decision makers rely on heuristics and thereby display several reasoning biases. At the same time, empirical data for the analytical Type 2 processes component show that, in healthy samples, impairments in analytical processing are observable both in clinical delusions [49,63] and as a correlate of paranoia [1,48] or false belief receptivity [57]. Only two studies reported a positive [54] or no association [64] of analytical processing with delusion occurrence. Regarding the JTC bias in particular, preliminary evidence suggests that premature decision-making is associated with less analytical processing [64] and linked to impairments in working memory, which is thought to be involved in analytical processing, thus aligning with the assumption that Type 1 and Type 2 processes interact in judgment and decision-making.

### Importance of the research question

In a nutshell, there is initial empirical evidence [48,49] and a growing body of theoretical conceptions [7,32,44] indicating that an aberrant Type 1/Type 2 processing interaction is associated with clinical delusions as well as non-pathological delusion-like experiences and possibly also with the JTC bias (see above). However, recent contributions have focused predominantly on diminished Type 2 processing in schizophrenia (e.g., [44]) and associated concepts of belief flexibility (e.g., [63]) and metacognition [65] and have rather ignored intuitive processing and its association with the JTC bias. Thus, many questions on intuition in individuals with schizophrenia and individuals experiencing non-clinical delusion-like experiences are still open. To our knowledge, apart from one cross-sectional study [49], there are no studies directly addressing the relationship between intuition and the JTC bias. Furthermore, prior studies have used cross-sectional designs applying mostly self-report questionnaires that do not allow inferences about the temporal relationship between intuitive/analytical processing and the JTC bias.

Therefore, we aim to close this research gap by specifically focusing on everyday life processing modes in schizophrenia and individuals with non-clinical delusion-like experiences, approximated by inspecting everyday life intuitive and analytical decision-making. In doing so, we aim at both studying the link between intuition/analysis and the JTC bias in a clinical sample (where patients with schizophrenia serve as a population that typically shows distorted information processing mechanisms, see for instance [38–41]) and in a non-clinical population sample as well. Examining a non-clinical sample thus contributes to the understanding of underlying mechanisms in aberrant reasoning in general and provides further evidence on a putative psychosis continuum. To circumvent the drawbacks of cross-sectional and laboratory-based designs adopted in previous studies, we will exploit an intensive, longitudinal approach by conducting an experience sampling methodology (ESM) study. Fortunately, technological innovations nowadays allow assessing mental phenomena and behavior in intensive observational designs *and* as they occur in real life [66]. In schizophrenia research, ESM approaches using smartphones have demonstrated to be feasible and reliable assessment modalities [67] with high compliance rates among patients [68,69] and in high-frequency sampling schemes [70], thus minimizing recall bias and allowing examination of temporally directed associations in natural settings [71,72]. The main research question is whether a prior high use of intuitive processing in everyday life predicts the subsequent occurrence of the JTC bias in patients with schizophrenia and in non-clinical individuals. Embedding our research into the dual-process frameworks of judgment and decision-making, our secondary research question is whether a prior low use of analytical processing in daily life predicts the subsequent occurrence of the JTC bias in patients with schizophrenia and in non-clinical individuals. Applying a psychosis continuum approach, we will also tentatively examine whether delusion proneness moderates the link between aberrant intuitive and/or analytical processing and JTC bias in individuals without a diagnosis of schizophrenia.

#### Proposed hypotheses

Addressing these research questions, we hypothesize that a prior high use of intuitive processing predicts subsequent JTC bias in patients with a schizophrenia disorder and in non-clinical individuals (Hypothesis 1). Also, aiming to replicate previous study results on delusion-related analytical reasoning [49] on a state level, we expect that a prior low use of analytical processing predicts subsequent JTC bias in patients with a schizophrenia disorder and in non-clinical individuals (Hypothesis 2). Given that intuitive processing emerges as a predictor of subsequent JTC bias in H1, we will also preliminarily test if prior intuition predicts subsequent paranoia in patients with a schizophrenia disorder (Hypothesis 3).

To investigate these associations on a psychosis continuum in individuals without a schizophrenia diagnosis, our fourth hypothesis is that the link between prior intuitive and/or analytical processing and subsequent JTC bias is moderated by delusion proneness (Hypothesis 4). A full description of these hypotheses along with sampling plans and intended analyses is provided in the Design Table (see Table 1 below).

**Table 1:** Design table.

Question	Hypothesis	Sampling plan (e.g. power analysis)	Analysis Plan	Interpretation given to different outcomes
*Primary research question*: Does a prior high use of intuitive processing in everyday life predict the subsequent occurrence of the JTC bias in patients with schizophrenia and in non-clinical individuals?	*Hypothesis 1*: A prior high use of intuitive processing predicts subsequent JTC bias in patients with schizophrenia and in non-clinical individuals.	It is planned to analyse data of *N* = 53 individuals with schizophrenia and N = 53 individuals without such a diagnosis. This estimate is based on assumptions of clinical feasibility and the lack of *apriori* parameter values that would allow the computation of effect sizes used for MLM-based power analysis. Accounting for a drop-out rate of 20%, we target to recruit *N* = 64 participants in this group. This sample size target serves as anchor point for all subsequent hypotheses too since–as mentioned–no informative effect sizes have been reported so far nor can be reasonably deduced.	*Main predictor*: Person-centered use of intuitive processing (range -4.99–4.99), where a higher value corresponds to greater intuitive processing compared to a person’s average intuitive processing. State negative affect will be entered as a control variable in order to control for potential confounding effects of negatively valenced affects. *Outcome measure*: JTC bias (possible values: 0, 0.5 or 1), where a higher value corresponds to a more pronounced jumping-to-conclusions bias (e.g., value of 0.5 means that a JTC bias was present in 1/2 scenarios). *Analytical strategy*: • Centered intuitive processing at prompt *t* will be entered as a predictor of JTC bias at prompt *t*. • Two multilevel models (cumulative link mixed models) with varying random effects will be tested. The first model will be specified with random intercept only, the other will also be specified with random slopes. The model with best model fit indices will be chosen for parameter interpretation, based upon a log likelihood ratio comparison. • If the final model yields a statistically non-significant coefficient (i.e., *p value* < .05), frequentist two one-sided equivalence tests (TOST) will be conducted for the corresponding coefficient. *Predicted direction of effect*: JTC bias will be significantly greater if centered intuitive processing at the same ESM prompt is more used (as opposed to less use).	*Outcome scenario 1*: If a high use of intuitive processing predicts a greater JTC bias in patients with schizophrenia, we interpret this as evidence that intuitive processing is positively related to subsequent JTC bias. *Outcome scenario 2*: If a high use of intuitive processing predicts a lower JTC bias in patients with schizophrenia, we interpret this as evidence that intuitive processing is negatively related to subsequent JTC bias. *Outcome scenario 3*: If a high use of intuitive processing does not predict JTC bias in patients in schizophrenia (i.e., *p value* < .05) and equivalence tests yield statistically equivalent effects, we interpret this as the expected effect of intuitive processing which we considered worthwhile being highly likely absent. *Outcome scenario 4*: If a high use of intuitive processing does not predict JTC bias in patients with schizophrenia (i.e., *p value* < .05) and equivalence tests yield effects that are not significantly equivalent (i.e., undetermined effect), we interpret this as the expected effect which we considered worthwhile being possibly present but statistically not detectable due to our study design (e.g., lack of statistical power).
	*Hypothesis 3*: *Given that intuitive processing emerges as a predictor of subsequent JTC bias in H1*, *intuitive processing predicts subsequent self-reported paranoia as a proxy for delusional symptoms*.	Analyses will be conducted with the patients with a schizophrenia spectrum disorder. Sample size calculations are based on the same premises as described in Hypothesis 1 (see Sampling Plan of Hypothesis 1).	Analyses for Hypothesis 3 will only be conducted if Hypothesis 1 is not fully rejected. *Main predictor*: Same main predictor as in Hypothesis 1. *Outcome measure*: State paranoia (range 1–5), where a higher value corresponds to higher levels of state paranoia. *Analytical strategy*: Same strategy as described in Hypothesis 1, with outcome state paranoia. *Predicted direction of effect*: State paranoia will be significantly greater if centered intuitive processing at the same ESM prompt is more used (as opposed to less use).	*Outcome scenarios*: Analogue to outcome scenarios 1–4 for Hypothesis 1.
*Secondary research question*: Does a prior low use of analytical processing in daily life predict the subsequent occurrence of the JTC bias in patients with schizophrenia?	*Hypothesis 2*: A prior low use of analytical processing predicts subsequent JTC bias in patients with schizophrenia and in non-clinical individuals.	Based on Sampling plan of Hypothesis 1.	*Main predictor*: Centered use of analytical processing (range -4.99–4.99), where a higher value corresponds to greater analytical processing compared to a person’s average analytical processing. *Outcome measure*: Same outcome measure as described in Hypothesis 1. *Analytical strategy*: Same strategy as described in Hypothesis 1, with main predictor centered analytical processing. *Predicted direction of effect*: JTC bias will be significantly greater if centered analytical processing at the prior ESM prompt is less used (as opposed to more use).	*Outcome scenarios*: Analogue to outcome scenarios 1–4 for Hypothesis 1.
*Tentative research question*: Does delusion proneness moderate the link between aberrant intuitive and/or analytical processing and subsequent JTC bias in individuals without a diagnosis of schizophrenia?	*Hypothesis 4*: The link between prior intuitive and/or analytical processing and subsequent JTC bias is moderated by delusion proneness in individuals without a diagnosis of schizophrenia.	Based on Sampling plan of Hypothesis 1. However, the analysed sample here refers to *N* = 53 individuals without a diagnosis of schizophrenia. Accounting for a drop-out rate of 20%, we target to recruit *N* = 64 participants in this group.	*Main predictors*: (a) Centered use of intuitive processing (range -4.99–4.99); (b) centered use of analytical processing (range -4.99–4.99); (c) trait delusion proneness as measured with the Peter’s Delusion inventory–total score (range 0–40), where a higher score corresponds to more delusion-like experiences. *Outcome measure*: Same outcome measure as described in Hypothesis 1. *Analytical strategy*: Same strategy as described in Hypothesis 1. Both centered intuitive and analytical processing will be entered as predictors into models. Each processing mode will be complemented by an interaction term with trait delusion proneness, in order to investigate the moderating role of trait delusion proneness. Additionally, two alternative models (one with random intercept only, one with random intercept and random slopes for both processing modes) which contain the covariable antipsychotic medication dose will be set up. This yields in total four models varying in the inclusion of the covariable and the specification of random effects, which will be compared to determine the best fitting model. *Predicted direction of effect*: JTC bias will be significantly greater if at the same prompt, centered intuitive processing is greater (as opposed to low use) and centered analytical processing is lower (as opposed to high use). If delusion proneness is enhanced, these effects will become more pronounced in the same direction.	*Outcome scenarios*: *Outcome scenario 1*: The link between centered intuitive processing and subsequent JTC is moderated by delusion proneness; this is not found for centered analytical processing. *Outcome scenario 2*: The link between centered analytical processing and subsequent JTC is moderated by delusion proneness; this is not found for intuitive processing. *Outcome scenario 3*: The link between centered intuitive processing and subsequent JTC is moderated by delusion proneness; an analogue moderation effect is also found for centered analytical processing. *Outcome scenario 4*: Neither the link between centered intuitive processing and subsequent JTC nor between centered analytical processing and subsequent JTC are moderated by delusion proneness. For scenarios 1, 2 and 4, in case of a non-significant frequentist predictor, equivalence tests may result in different scenarios ((a) statistically equivalent effect; (b) non-significant equivalent effect, i.e., undetermined effect). Sub-scenario (a) will be interpreted as evidence that the effect of interest considered worthwhile is highly likely absent. Sub-scenario (b) will be interpreted as evidence that the effect of interest is possibly present but probably not detectable with frequentist statistics.

Note. This table includes planned analyses as well outcome-contingent analyses (e.g., equivalence tests). Further tests deemed reasonable as post-hoc tests will, if conducted, be transparently communicated as unregistered analyses.

JTC = Jumping-to-conclusions, MLM = Multilevel model.

To our knowledge, our hypotheses have not so far been tested in an intensive longitudinal design using a state operationalization of intuitive and analytical information processing. By addressing our research questions, we aim to further improve the understanding of putative delusion-underlying mechanisms of schizophrenia disorders. In the light of a putative psychosis continuum, we also expect to extend findings to a non-clinical sample and thus contribute to the identification of proximate factors underlying human aberrant reasoning. It is hoped that this study will not only advance the theoretical understanding within the framework of the Dual Stream Modulation Failure model [7,73], but, in the long term, may also inform clinical intervention. Showing that temporally preceding processing modes are linked to subsequent JTC bias and paranoid conviction could imply that interventions aiming to reduce clinically relevant delusions may also benefit from focusing more on processing modes rather than on the JTC bias alone (see [74] for an example of a pioneering clinical trial on this endeavor). Moreover, general information-processing models (e.g., [2]) will also be informed by our results.

## Methods

### Ethics information

This intensive longitudinal study forms part of a bigger project on “Intuition and jumping to conclusions in schizophrenia: Studying the temporal relationship in daily life and with neuroimaging” (SNSF grant number: 179897, see http://p3.snf.ch/project-179897). We registered the study at the Open Science Framework (see https://osf.io/8gkjx/). The study protocol and all data will be made publicly available there.

The study has been approved by the competent ethics committee and complies with all relevant ethical regulations. We will only assess participants who have provided informed consent. For patients, capacity to consent will be determined by the attending psychiatrist/psychologist. Each participant will be financially compensated with a money-equivalent local voucher, equating 130 Swiss Franc per person in total (including two fMRI sessions à 40 CHF each, maximum 12 ESM prompts à 2 CHF each prompt, a bonus of 5 CHF if at least ten of twelve ESM prompts are completed, and one clinical interview à 20 CHF).

### Sampling plan

#### Participants

Inclusion of 106 participants is planned. We target to recruit a total of 128 participants, allowing for a dropout rate of 20% (e.g., due to non-compliance), and will analyze data of all participants who provide valid data and fulfill all inclusion criteria. Half of the participants (*N* = 64) will be patients with a diagnosed ICD-10 schizophrenia disorder recruited from the local psychiatric university hospital. Diagnoses of patients will be based on diagnostic assessments conducted by trained psychologists or psychiatrists as part of the hospital admission process and will be derived from electronic records by the research staff (antipsychotic medication dose will also be derived from these records). Patients will be recruited both from psychiatric inpatient wards and from one outpatient ward of the local psychiatric university hospital. The other half of the participant sample (*N* = 64) will be individuals from the general population without a diagnosis of schizophrenia.

We did not conduct power analyses for the study at hand for the following reason: Given the multilevel study design, the nested data structure needs to be considered when calculating the optimal sample size *a priori*. Due to a current lack of intensive longitudinal studies on intuition in schizophrenia, we cannot make reliable assumptions regarding most of the important multilevel model parameters such as random predictor variances and covariances (as suggested by [70]), which, however, is indispensable when running a simulation study for nested data designs. In consultation with those of our co-authors working in clinical practice, our sample size decision is hence based upon the premise of clinical feasibility, and accounts for the rather low prevalence rates of schizophrenia disorders and the need to secure study feasibility (e.g., recruitment issues) within an adequate time span.

#### Inclusion and exclusion criteria

Inclusion criteria for individuals with and without schizophrenia are: (a) fluent German, (b) IQ >70, (c) normal or corrected-to-normal vision, (d) age between 18 and 55 years, and (e) an ESM compliance rate for fixed prompts of at least 33% (following procedures in other ESM studies, e.g., [75]). An additional inclusion criterion for the patients is (f) the presence of a primary ICD-10 diagnosis of either schizophrenia, schizotypal, delusional, or another non-mood psychotic disorder (F20-F29) with clinically relevant delusional symptomatology. The inclusion criteria for the patients are relatively broad in scope in order to enhance generalizability of the results. Exclusion criteria for all participants are: (a) acute suicidality, (b) current neurological diseases, (c) current or past primary diagnosis of substance dependence, and (d) non-fluent German, and (e) if major technical issues occur (e.g., lost smartphones or smartphones not working reliably after start of ESM interval). Participants will also be excluded under this criterion if triggering problems with the ESM software should occur repeatedly, such as more than 67% of all prompts not being triggered and thus impairing participants technically from achieving a minimum compliance rate of 33% (which we do not expect, but hypothetically itemize). Additional exclusion criteria for the participants from the general population are: (f) current or past axis I mental and/or neurocognitive disorders, (g) current or past schizophrenia, schizotypal, and delusional disorders, and bipolar affective disorders in first-degree relatives (to exclude participants with a genetic psychosis risk).

### Design

#### Procedure and materials

Adult individuals without a diagnosis of schizophrenia will be recruited via public advertisements and will undergo a pre-screening telephone interview with one author in order to assess their eligibility for study participation. Patients considered eligible for the study will be approached by the same author and, if interested, invited for a laboratory assessment. During the laboratory assessment, both individuals without a diagnosis of schizophrenia and patients will be provided with detailed study information, informed consent procedures will be conducted, and first trait assessments will be undertaken (described below). These trait assessments will take place in order to rule out the presence of any current or lifetime schizophrenia or psychotic disorder and to capture potential delusion ideation in participants without a diagnosis of schizophrenia, to validate the life-time presence of a psychotic disorder and determine levels of psychopathology severity in patients, and to obtain measures of trait decision-making preferences and fluid intelligence (as a proxy for general intelligence) across all participants (the latter two not relevant for the study at hand). If considered fully eligible, participants will be invited for a second appointment within the next seven days where they will be trained to charge and use ESM devices that will then be handed out to participants, and a turnover appointment for the week after the last ESM prompt will be arranged.

Participants will be handed out smartphones in order to participate in the ESM sampling interval of three consecutive days. We will provide Android smartphones that have proved to be compatible with the chosen ESM web-based software movisensXS, Version 1.5 (movisens GmbH, Karlsruhe, Germany). All smartphones handed out to participants will be equipped with the ESM app movisensXS, which will automatically trigger prompts from sampling day 1 onwards until the end of sampling day 3. The devices will be pre-adapted by the research team instructor so that participants can only change minor study settings such as font size but will be unable to interfere with the study settings itself. If participants themselves own smartphones of the models specified above and wish to use these to participate in the ESM study, they will be supported in installing the movisensXS application on their personal devices.

Smartphone prompts will be triggered four times a day (10 a.m., 1 p.m., 5 p.m., 9 p.m.), resulting in a total of 12 prompts, and can be answered, postponed, or dismissed. Each assessment will take around 5–6 minutes. This sampling scheme is chosen to reduce and distribute study participation burden for schizophrenia inpatients who frequently experience severe illness-related difficulties in participating (e.g., concentration difficulties) and to enhance adherence to the ESM procedure. The time frames for prompts were chosen so as to ensure that the four prompts can be responded to during waking hours and that the four prompts appear at comparable intervals. The arrival of each prompt will be announced by a beep, and answering the prompt may be delayed by a maximum of 20 minutes only, as it has been suggested that the level of reliability of delayed reports diminishes fairly rapidly [76].

In order to enhance compliance, the instructor will train with each participant the completion of one representative ESM prompt and answer any related questions. Regarding remuneration procedure, all participants will be compensated proportionally to the amount of completed ESM prompts (2 Swiss Francs per prompt). To further increase compliance, the research team instructor will contact participants after the first day of ESM participation in person (patients), or else via the app-in-built chat, to inquire after potential problems or unclear points.

#### Baseline assessments

Demographic assessments will comprise questions regarding age, gender, education level, and housing situation and will be designed by the research team. Patients with schizophrenia will also undergo a psychopathology rating (external assessment administered by a person from the research team).

In order to confirm the absence of any major Axis I DSM-IV mental disorder in non-clinical individuals, including a schizophrenia spectrum disorder, all individuals from the general population will undergo a structured clinical interview, the Mini International Neuropsychiatric Interview (MINI, [77]). Individuals who fulfill a prior and/or present diagnosis as assessed with the MINI are subsequently excluded from the study. For the group of individuals with a diagnosis of schizophrenia, the diagnosis given by the local university hospital ward will be confirmed with the sections for depression, mania and psychotic disorders of the MINI. All interviews will be conducted by trained members of the research team who regularly receive diagnostic supervision.

Delusion proneness in individuals without a diagnosis of schizophrenia will be assessed using the Peters Delusion Inventory (PDI; [78]) in its validated German translation (79). In 40 items, this inventory assesses present delusion-like beliefs of an everyday life nature instead of clinical symptoms. Each item (e.g., “Do you ever feel as if people are reading your mind?”) is answered in a dichotomous format (yes/no), and items answered with ‘yes’ yield 5-point Likert scale items regarding the levels of distress, preoccupation, and convictions linked to the belief (ranging from 1 *= not at all distressing*, *hardly ever think about it*, *do not believe it’s true*; to 5 *= very much*, *think about it all the time*, *believe it’s absolutely true*). This instrument has in its original version been used in various studies examining non-clinical populations (e.g., [80]) and demonstrates acceptable indices of reliability [78] and good validity [81]. The same holds true for the validated German translation [79]. Each participant will complete further assessments/testings that are, however, not of relevance for this study.

#### ESM assessments

A detailed description of all ESM items used in this study is listed in S1 Table in S1 Appendix. Notably, while our hypotheses will be addressed inspecting predictors and outcomes always measured within the same prompt, the nature of our study is longitudinal since information processing modes are assessed with respect to an actual everyday-life decision of the participant “since the last prompt”and JTC bias is assessed “at the moment” (measures see below).

Intuitive and analytical processing (predictors) will be assessed on a state level with a short and state-adapted version of the REI-40 ([26], for the adapted version, see Supplementary material S1 Appendix) that was recently developed and validated with the aim of being used specifically in ESM designs. This state-adapted version will be used to approximate intuitive and analytical processing with regard to an actual everyday life decision of the respective participant. Like prominent self-report instruments in the field of dual process theories, we will target intuitive and analytical processing by focusing on self-reported decision-making as an observable proxy for Type 1 and Type 2 processes. Items were chosen based on differential factor loadings reported in REI-40 studies [26,82]; the full development strategy is reported elsewhere (see S1: [1]).

The JTC bias (outcome) will be assessed with an adapted version of the Fictitious Scenario Task designed by Lüdtke and colleagues [83]. This task is a JTC-adapted, close-to-everyday-life version of a measure originally created to assess the bias against disconfirmatory evidence. Moritz and colleagues [84] could show that this JTC-adapted version is as appropriate as classic JTC measures, such as the Beads Task to measure jumping to conclusions. At first glance, the scenarios in the Fictitious Scenario Task can be interpreted in different ways and are thus ambiguous (see S1 Fig in S1 Appendix for example item). Participants will be instructed to give a confidence rating on the validity of the four given explanations in the light of a first sentence (response options: 0%, 25%, 50%, 75%, or 100%). Subsequently, two further sentences will be revealed that may change the plausibility of presented explanations and for which new confidence ratings can be given (not of interest in this study). With each revealed sentence, participants can also make a final choice for any explanation given. As our primary JTC bias outcome, confidence ratings ≥ 75% after the first sentence are considered as hasty decision-making and indicative of the JTC bias. An alternative JTC bias outcome can be computed contingent on whether the primary outcome gives rise to a floor effect and does not display enough variability to set up statistical models or not: If not, a “final decision” statement made after sentence 1 (too early to make a valid judgment) is considered as hasty decision-making and indicative of the JTC bias present at the moment of the testing.

Paranoia, as a proxy for delusional symptoms in patients with schizophrenia, will be assessed with the 5-item brief state version of the Paranoia Checklist [85].

These ESM assessments as well as the assessment of state negative affect (see Table 1) will be completed by each participant at each prompt, while reactivity (see Table 1) will be assessed only at the last prompt of the day.

### Analysis plan

#### Pre-processing

Raw data from all participants will be obtained via the movisens webbased platform by one member of the research staff (SK) and will be cleaned and pre-processed before main analyses: First, raw laboratory assessment files and raw ESM data will be merged by a unique participant identifier. Second, all prompts that have not been filled out completely or have been dismissed will be treated as missing prompts. If unique ESM data points relevant to answering the main hypotheses are missing, the corresponding prompts will not be included in analyses relying on the respective data. If due to any (unexpected) technical distortions more prompts than planned, or theoretically impossible values (e.g., repeated prompts or inputs exceeding predefined input ranges), were stored, the data values will be checked and removed if necessary. Third, all individuals with a compliance rate for all fixed prompts below 33% will be excluded, as it will otherwise be difficult to maintain the assumption that data are missing completely at random [72].

Next, we will also check if main outcomes are normally distributed and, if this is not given, test if transformations can help to normalize data distributions. If transformation does not change the distribution statistically significantly as indicated by a Shapiro Wilkes test, we will keep the raw data for analyses. Lastly, we will also remove outliers from the corresponding variables, defined as values lower or higher than the mean ± 3 standard deviations; we will not remove outliers detected in major ESM variables, as removing outliers from these brief Likert scales might distort the data distribution substantially.

Due to the hierarchical structure of our data, where different observations (level 1) are nested within individuals (level 2), we will employ multilevel model analyses. As we aim to obtain regression estimates of level-1 associations that are unambiguously interpretable, we will person-mean center all time-varying predictors by subtracting the cluster’s (i.e., person’s) mean predictor value from the given predictor value. We thus follow the recommendations by [86] who have advised person-mean centering if a level-1 association is of substantial research interest, thereby obtaining a fairly accurate coefficient estimate unaffected by level-2 variable influences. As an example, a novel variable “intuitive processing centered” will be created with values that are centered around zero and that represent deviations in the predictor from an individual’s average predictor value: Positive values represent a more-than-usual intuitive processing for this individual, negative values represent a less-than-usual intuitive processing for this individual. For predictor and outcome variables, we will also compute person-mean variables for descriptive reporting.

#### ESM analyses

As part of our preliminary analyses, we will compute ESM-related measures of compliance. To this end, we will derive each person’s mean number as well as corresponding standard deviations of completed prompts (individual compliance rates) and group-specific compliance rates. As done in other studies in the field of clinical psychology [67,69], participants will only be counted as “completers” and included in the study if they have completed at least 33% of all fixed prompts [72]. We will also test in an exploratory manner whether completers and non-completers differ significantly in demographic variables, psychopathological severity rating, state negative affect, or with regard to the ratio of individuals with and without a diagnosis of schizophrenia, using *t*-tests for independent samples or Mann-Whitney U tests (if data are not normally distributed), and chi square tests or Fisher’s exact tests (if any cell has < 5 observations). In order to assess to which degree reported information processing modes were “as usual”, we will also address to which degree the ESM procedure itself may have influenced reporting of processing modes by increasing self-awareness (denoting a reactivity phenomenon): We will tentatively examine the relevance of ESM reactivity for processing modes by regressing participants’ raw day-averaged information processing modes separately onto this day’s reactivity score (derived by averaging both reactivity item scores, see Supplementary material Table 1). All further descriptive and inference statistical tests, including primary analyses, will only be computed with completers.

All statistical pre-processing and analyses will be computed using R (Version 4.0.0 or later; [87] and will use a significance threshold of α = 5%.

#### Primary analyses

As described above, our ESM data structure is of a hierarchical structure where it is assumed that observations within clusters (here: individuals) are not independent, thus requiring the use of multilevel models (MLM). Intra-class correlation (ICC) coefficients will be computed for major predictors and outcomes to obtain an estimate of between-person and within-person variances. We will use cumulative link mixed models [88], as our outcome variables are both assessed on an ordinal scale (JTC: three levels; self-reported paranoia: five levels). All MLMs will contain a random intercept and for each time-varying predictor an additional random slope if this improves model fit (as indicated by log-likelihood ratio statistics), thereby considering model complexity.

For H1, H2, and H3, we will refrain from including time-invariant covariates like age, sex, or antipsychotic medication dose as all these cannot have an influence on the estimated relationship between our time-varying predictor and outcome when the time-varying predictor is person-centered [89]. We will, however, include state negative affect as time-varying covariate in all models in order to control for potential confounding effects. For H4, which involves delusion proneness as time-invariant moderator, we will set up two different models, one with and one without antipsychotic medication dose as time-invariant covariate to control for. In the model with the covariate, we plan to enter the predictor antipsychotic medication dose as well as an interaction term of *dose x delusion* proneness (our original moderator). Again, the best fitting model for H4 will be determined based on log likelihood ratio test statistics. For each hypothesis, one final model will be used to derive relevant coefficients and other interpretable parameters.

For hypothesis H1, we will test whether prior intuitive processing (referring to an actual decision “since the last prompt”) predicts subsequent JTC bias (referring to a JTC bias assessed “at the moment of the prompt”). For hypothesis H2, we will test whether prior analytical processing (referring to an actual decision “since the last prompt”) predicts subsequent JTC bias (referring to a JTC bias assessed “at the moment of the prompt”). For hypothesis H3, we will test whether prior intuitive processing (referring to an actual decision “since the last prompt”) predicts subsequent paranoia (referring to state paranoia assessed “at the moment of the prompt”). For hypothesis H4, we will test whether prior intuitive processing and/or analytical processing (referring to an actual decision “since the last prompt”) predict subsequent JTC bias (assessed “at the moment of the prompt”) and whether these associations will vary with the level of trait delusion proneness (interaction between trait delusion proneness and intuitive processing and/or analytical processing). For each MLM, we will also run diagnostic checks to assess model assumptions, including the proportional odds assumption for ordinal outcomes. Full model specifications of all final models as well as assumptions on random effect variances will be provided in the supplemental material.

#### Contingent analyses

Regarding Hypothesis 1–4, we plan to compute frequentist equivalent tests relying on two one-sided tests (TOST) if a predictor or interaction term in the null hypothesis significance tests does not turn statistically significant as theoretically reasoned. If we find that intuitive processing predicts subsequent paranoia as a proxy for delusional symptoms in patients with schizophrenia, we will also preliminarily investigate if intuitive processing predicts subsequent hallucinations.

Further post-hoc tests may become reasonable after main analyses, although not anticipated a priori; these post hoc analyses will then be transparently justified and reported in accordance with pre-registration recommendations [90].

### Proposed timeline

We will take up data collection as soon as possible after Stage 1 acceptance (anticipated in summer 2021). We aim to complete data collection within 26 months (until end of summer 2023), and conduct data analysis and finalize our stage 2 manuscript within 3 months.

## Supporting information

S1 Appendix A. References for unpublished or submitted word. B. Figure S1: ESM Fictitious Scenario Task. C. Table S1. ESM items.
(DOCX)
