# Supplementary material for: Is intuition allied with jumping to conclusions in decision-making? An intensive longitudinal study in patients with delusions and in non-clinical individuals
Source: PLoS One. 2021 Dec 20;16(12):e0261296. doi: 10.1371/journal.pone.0261296 (PMC8687575; doi:10.1371/journal.pone.0261296)
Supplement: S1 Appendix — A. References for unpublished or submitted word. B. Figure S1: ESM Fictitious Scenario Task. C. Table S1. ESM items. (DOCX) [file pone.0261296.s001.docx]

# Supplementary methods

**Stage 1: Supplemental material**

1. **References for unpublished or submitted work**
2. Zander-Schellenberg T, Kuhn SAK. Intuition and reflective analysis in daily life – Introducing a short-scale version of the Rational-Experiential Inventory to assess decision-related information processing at a state level. Submitted manuscript.

1. **Figure S1: ESM Fictitious scenarios task**


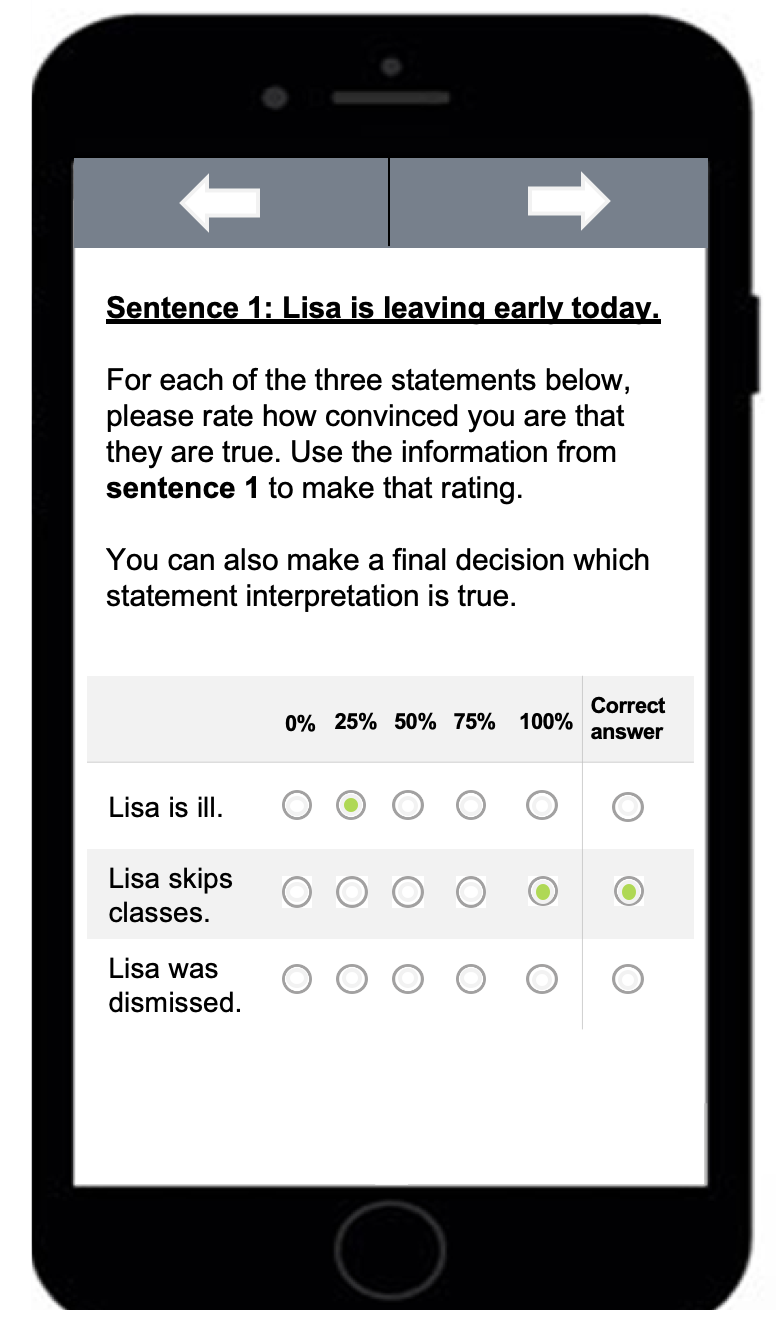


1. **Table S1: ESM items**

| Construct | | Number of items | ESM measure | Response format |
| --- | --- | --- | --- | --- |
| Negative affect |  | 5 | State negative affect will be assessed similar to previous ESM studies assessing negative affect^1-3^, and is used as a covariate. Participants are asked to indicate their *momentary* levels of anxiety, insecurity, loneliness, guilt feeling, and sadness. Example: “How anxious do you feel at the moment?”^a^ | 7-point Likert scales  (from 1 = “not *X* at all” to 7= “very *X*”). |
| Psychotic symptoms | State paranoia | 5 | State paranoia will be assessed with the 5-item brief state version of the Paranoia Checklist, which was validated for the German language (BSPC^4^). Participants are asked to indicate how much they are convinced *at the moment* that the 5 statements are true: Example: “Peoply try to make me upset.” | 5-point Likert scales (from 1 = “not convinced at all” to 5 = “very much convinced”) |
|  | State hallucinations | 1 | State hallucinations will be assessed with one self-developed item:  “Do you have a sensory experience at the moment that seems strange/not real for you?” | Dichotomous answer format (“yes” vs. “no”) |
| Information processing mode | Decision area | 1 | Participants are asked to identify one actual *decision made since the last prompt*. Participants can ascribe their taken decision to one or more life domains, namely: (1) education or work, (2) friends or leisure time, (3) romantic relationship or family, (4) health and nutrition, (5) consumer behavior or (6) other. | Dichotomous answer format (“yes” vs. “no”) |
|  | Intuitive processing | 4 | The Faith in intuition (FI) subscale of a novel state-adapted inventory designed to assess decision-related information processing modes in ESM designs^5^ will be used to capture intuitive processing^b^. Participants will be asked to indicate how much each of four statements applied to them *since the last prompt*. Example: “I liked to rely on my intuitive impressions.” | 5-point Likert scales (from 1 = “does not apply to me at all” to 5 = “very likely applies to me”) |
|  | Analytical processing | 4 | The Need for cognition (NFC) subscale of the above-mentioned state-adapted inventory^5^ will be used to capture analytical processing^b^. Participants will be asked to indicate how much each of four statements applied to them *since the last prompt*. Example: “I enjoyed solving problems that required hard thinking.” |  |
| JTC bias | Primary outcome | – | Per prompt, two fictitious scenarios will be presented for which up to two hasty decisions in total can be made which scores are averaged, resulting in a JTC score of either 0 (no JTC), 0.5 (JTC in one scenario) or 1 (JTC in both scenarios) per prompt. In few random prompts, one scenario will be a filler scenario where the initially plausible explanations remain plausible until the end in order to avoid insight into the nature of the task; here, only JTC scores of 0 or 1 can be obtained. | – |
|  | Alternative jtc outcome | – | Analogous to the primary jtc outcome, an averaged JTC score of either 0, 0.5 or 1 per prompt can be obtained. |  |
| Reactivity |  | 2 | End-of-day reactivity will be assessed using two items from a prior ESM study in this field^6^: ”To what extent did completing these questionnaires influence your mood today?”  ”To what extent was this an ordinary day for you?” | 0 to 100 slider scale (0 = “no influence at all”/”very ordinary day”, 100 = “very strong influence”/”very unordinary day”) |

^1^ [2]; ^2^[3]; ^3^ [4]; ^4^ [5]; ^5^ [1]; ^6^ [6].

^a^ An average score of the five items is used as an approximator for state negative affect.

^b^ A total score for FI and NFC subscale, respectively, will be derived by summing up their two-item subscales “intuitive ability” (or “analytical ability”, respectively) and “intuitive engagement” (or “analytical ability”, respectively).

**References Supplementary Information**

1. Oorschot M, Lataster T, Thewissen V, Lardinois M, Wichers M, Van Os J, et al. Emotional experience in negative symptoms of schizophrenia-no evidence for a generalized hedonic deficit. Schizophr Bull. 2013; 39: 217–25.
2. Ruan Y, Reis HT, Zareba W, Lane RD. Does suppressing negative emotion impair subsequent emotions? Two experience sampling studies. Motiv Emot. 2019; <https://doi.org/10.1007/s11031-019-09774-w>
3. Reininghaus U, Kempton MJ, Valmaggia L, Craig TKJ, Garety P, Onyejiaka A, et al. Stress sensitivity, aberrant salience, and threat anticipation in early psychosis: An experience sampling study. Schizophr Bull. 2016; 42: 712–22.
4. Schlier B, Moritz S, Lincoln TM. Measuring fluctuations in paranoia: Validity and psychometric properties of brief state versions of the Paranoia Checklist. Psychiatry Res. 2016; 241: 323–32. <https://doi.org/10.1016/j.psychres.2016.05.002>
5. So SH, Peters ER, Swendsen J, Garety PA, Kapur S. Detecting improvements in acute psychotic symptoms using experience sampling methodology. Psychiatry Res. 2013; 210: 82–8. <https://doi.org/10.1016/j.psychres.2013.05.010>
